# Bacterial Vaginosis [BV] knowledge, attitudes and behavioural changes after BV diagnosis among women enrolled in a clinical trial in Boston, USA and Vulindlela, South Africa: a qualitative study

**DOI:** 10.1186/s12905-025-04210-3

**Published:** 2025-12-12

**Authors:** Cecilia Milford, Buyisiwe L. Dlamini, Nomfuneko A. Mafunda, Timothy S. Hardwick, Lara Lewis, Anam A. Khan, Makhosazane N. Mdladla, Caroline M. Mitchell, Disebo Potloane

**Affiliations:** 1 Centre for the AIDS Programme of Research in South Africa (CAPRISA), Vulindlela, South Africa; 2 Wits MRU (MatCH Research Unit), Department of Obstetrics and Gynaecology, Faculty of Health Sciences, University of the Witwatersrand, Durban, South Africa; 3 SYNAPSE, School of Clinical Medicine, Faculty of Health Sciences, University of the Witwatersrand, Pietermaritzburg, South Africa; 4 Vincent Center for Reproductive Biology, Massachusetts General Hospital, Boston, MA USA; 5 Wits RHI, Faculty of Health Sciences, University of the Witwatersrand, Johannesburg, South Africa

**Keywords:** Bacterial vaginosis, Knowledge and attitudes, Health seeking behaviour, Vaginal hygiene, USA, South Africa, Qualitative research

## Abstract

**Background:**

Bacterial vaginosis (BV), the most common cause of vaginitis among reproductive age women, has high recurrence even after treatment. In addition to the physical risks of BV (e.g. increased HIV risk and adverse pregnancy outcomes such as preterm birth), the symptoms (malodour, itchiness, vaginal discharge) can cause emotional impacts (e.g. shame, embarrassment, self-consciousness). BV knowledge, attitudes, behavioural change(s) after diagnosis and other previous vaginal health concerns were explored among participants in a clinical trial assessing the safety and biologic effects of a vaginal live biotherapeutic product.

**Methods:**

In-depth interviews (IDIs) were conducted with 37 female participants in a clinical trial in Boston, USA (*n* = 14), and Vulindlela, South Africa (SA) (*n* = 23). Interviews were audio recorded, transcribed and translated. Transcripts were coded and thematically analysed using NVivo.

**Results:**

In this study, previous BV diagnosis was lower in SA than USA (2(9%) versus 12(86%)), with 7(19%) overall reporting a previous STI. There were five key themes: (1) Prior BV and other vaginal health experiences: Although BV was not frequently diagnosed in SA, participants reported experiencing symptoms (discharge, itchiness and malodour). Conversely, USA participants had experiences with previous BV diagnoses. (2) Previous vaginal health seeking behaviour and treatments: Almost all participants from both sites had sought treatment from healthcare professionals for vaginal health concerns, with a few from both sites having used self-care practices. (3) BV knowledge, attitudes and reactions to BV diagnosis: BV knowledge in SA was poor compared with the USA, but most participants were relieved to receive help. (4) Behavioural changes because of BV: Vaginal hygiene practices changed and participants washed more frequently/used products to address malodour. Further, BV symptoms resulted in changes in sex acts and/or abstinence. (5) BV diagnosis disclosure patterns: Disclosure varied, with participants commonly disclosing to those who could provide them with support.

**Conclusions:**

BV knowledge, attitudes and experiences are related to previous diagnosis and treatment. Symptoms impact individuals’ behaviours regardless of previous diagnosis, and can have psychosocial impacts which need to be addressed via appropriate counselling and treatment strategies. Ongoing research for effective BV prevention and treatment options that can be made available and accessible to women with BV globally is needed.

**Trial registration:**

South African National Clinical Trials Registry (SANCTR DOH-27-102023-8342; October 27, 2023) and ClinicalTrials.gov (NCT06135974; November 02, 2023).

**Supplementary Information:**

The online version contains supplementary material available at 10.1186/s12905-025-04210-3.

## Background

Bacterial vaginosis (BV) is the most common vaginal disorder in women of reproductive age worldwide [1–4]. It is a disruption of the vaginal microbiota, where there is a shift from *Lactobacilli* dominance to a diversity of anaerobic bacteria [1]. Globally, the general population prevalence of BV is high. One systematic review found a BV prevalence of 27% in North America and 25% in Sub-Saharan Africa [5]. A more recent systematic review in pregnant women in Sub-Saharan Africa found a higher prevalence, ranging from 28.5% in Eastern Africa to 52.4% in Southern Africa [6]. In young South African women aged 15–24, a separate estimate for BV prevalence was 42.1% [7].

BV is associated with various adverse health outcomes in those affected, including increased risk of sexually transmitted infections (STIs) and HIV, pelvic inflammatory disease, urogenital infections, and abnormal pregnancy or adverse pregnancy outcomes such as risk of preterm birth [1, 5, 8]. BV clinical manifestations range from asymptomatic to increases in vaginal discharge and experiences of vaginal malodour [1, 8–10].

In addition to the physical symptoms of BV, women may experience negative psychosocial impacts which can adversely affect their quality of life [4, 8, 11]. These multi-dimensional (physical and emotional) impacts of BV and BV symptoms have led to poor self-esteem, with feelings of shame, embarrassment and self-consciousness in many women [8, 11]. Further, concerns about malodour have resulted in avoidance of some sexual practices, and even abstinence, affecting relationships between sexual partners [8, 11]. In addition, fear that others may detect their abnormal odour or discharge [12] has impacted women’s social and work lives, which can have economic implications.

BV is usually treated with antibiotics, such as oral or intravaginal metronidazole and clindamycin [4, 11], with varying rates of recurrence after treatment. In the United States of America (USA), people are treated based on diagnostic tests performed in clinics or a laboratory [13, 14]. In South Africa (SA), the Primary Healthcare Standard Treatment Guidelines [15] recommend syndromic management of vaginal discharge, without routine screening for the cause of discharge. This includes BV treatment as part of a treatment package for all potential causes of vaginitis [15]. There are reports of BV recurrence rates of more than 50–69% within 12 months of treatment [4, 16, 17]. As a consequence, alternative strategies such as intravaginal boric acid [4] and other self-help or traditional methods such as yoghurt or probiotics, have been used by some women to treat their BV [3].

Affected women have attributed their BV acquisition and recurrence to sexual activities in general, including unprotected sex and sex with a new male partner [12, 18, 19]. Lifestyle factors such as stress, have, to a lesser extent, also been attributed to increased risk of BV [18, 20]. In addition, personal hygiene, such as soaps used to wash the vagina, have been viewed as triggers by women who have BV [19] resulting in changes in vaginal hygiene practices to manage BV.

This manuscript explores the BV related knowledge and attitudes of participants in a clinical trial of a novel vaginal product to reduce recurrent BV (VIBRANT - Vaginal lIve Biotherapeutic RANdomized Trial). In addition, any behavioural change or outcomes experienced by these participants after a BV diagnosis are explored. Furthermore, vaginal health concerns and/or conditions prior to enrolment in the clinical trial and the relationship between this and participants’ BV knowledge and attitudes during the clinical trial, are explored. Understanding these factors is important to be able to identify public health interventions which can support individuals with BV to have improved quality of life.

## Methods

### Study setting

This qualitative study was a sub-study of the VIBRANT study (full title: Vaginal Live Biotherapeutic Randomised Trial: Phase 1 trial of multi-strain *Lactobacillus crispatus* vaginal live biotherapeutic product (LBP)). VIBRANT was a phase 1 clinical trial that assessed the safety and biologic effects of two LBPs in women with BV (determined by Amsel and/or Nugent score). All participants in the clinical trial had BV, were treated with oral metronidazole for seven days, and were then randomised to one of five groups. For all five groups it was required that a vaginal tablet (either placebo or a variation of the LBP) was inserted intravaginally for seven days. The timing and composition of the LBP tablets varied slightly between the trial arms [21].

Both the VIBRANT study and qualitative sub-study were conducted in Boston, Massachusetts in the USA, and Vulindlela, KwaZulu-Natal in South Africa. Boston is a city of approximately 650,000 in a metropolitan area of over 4 million people, and is home to a large number of colleges and universities [22]. The Boston study site is in a private hospital research clinic. Vulindlela is a rural area, consisting largely of *isiZulu* speaking people, high levels of poverty, poor infrastructure and high HIV prevalence [23]. Healthcare is accessible to community members through public health facilities located in this area. The Vulindlela study site is located in a research centre adjacent to a public health clinic.

The VIBRANT study was conducted from February 2024 to February 2025 [21] and the qualitative sub-study was conducted concurrently from February to December 2024. The overall aim of this qualitative study was to understand and explore the experiences and preferences of women using a vaginally delivered LBP in the clinical trial to inform future product development. One component was to specifically explore knowledge, impact and outcomes of a BV diagnosis, which is the focus of this manuscript.

### Qualitative sub-study: population and data collection

Qualitative sub-study participants were aged 18–40 years, and were purposively selected from those participating in the VIBRANT study. They all had BV (an inclusion criteria at enrolment for the VIBRANT study), and as part of the clinical trial their BV was treated by metronidazole followed by vaginal insert (LBP/placebo) use. This allowed us to explore knowledge and attitudes of people who had been diagnosed with BV (either previously or during the study).

We aimed to select a representative sample of approximately 15–20 participants per study site (30–40 in total), representing a range of participant ages and product use experience. All of these participants were invited to participate in a series of two in-depth interviews (IDIs) at two different timepoints in the clinical trial. The first IDI was to be conducted at, or soon after product use in the clinical trial, and the second three weeks later, to determine if there had been any changes over time.

In addition, we aimed to recruit about 5–10 participants for once-off cases of interest IDIs which could be done at any time point during the study. These IDIs included participants from the VIBRANT study who had note-worthy experiences during their participation in the clinical trial, including challenges with product insertion or adherence, early discontinuation of product, or those who were eligible to participate but chose not to participate.

The IDIs had semi-structured interview guides developed specifically for this study (see supplementary files), and explored user experiences, product acceptability, facilitators and barriers to product adherence (and use) and the acceptability of an applicator for product insertion. The knowledge, emotional impact, and behavioural outcomes of a BV-related diagnosis and treatment were also explored. This latter thematic area is the focus of this manuscript.

All IDI participants provided separate written or verbal consent to participate in the qualitative sub-study as per requirements of site-specific ethics boards. The IDIs were approximately 1–1.5 h in duration, and were conducted in a quiet, private venue, at a time that was convenient to the participant. In Vulindlela, South Africa, interviews were conducted in person, and in Boston, USA, they were conducted over an online platform (such as Teams or Zoom) or in person depending on participant preference. Interviews were conducted by interviewers trained both in qualitative research and in sensitive research topics. In South Africa, all interviewers were female, bilingual (first language *isiZulu* speakers), who were of the same race and cultural background as participants at the South African site (BLD, MNM and one other interviewer). All interviews at Vulindlela were conducted in *isiZulu*, the home language of the participants. In the USA, all interviews were conducted in English by a female researcher from the USA (NAM), which enabled an understanding of and ability to reflect on the context of the USA participants. The interviews were audio-recorded, with participants’ consent.

Demographic and relevant quantitative behavioural data were collected as part of the VIBRANT study’s clinical component; for example, baseline behavioural sexual and reproductive health data, such as BV and sexually transmitted infections (STIs), were collected (see supplementary files for study specific baseline behavioural questionnaire).

### Data analysis

Relevant clinical trial demographic and baseline behavioural data were descriptively analysed using medians, interquartile ranges (IQR), percentages and frequencies in SAS, version 9.4 (SAS Institute Inc). These data were used to describe participants’ sociodemographic characteristics and individual-level BV and STI history.

IDIs were transcribed and translated into English (where necessary). Once transcribed, the SA study team (CM, TSH, BLD, MNM) developed a single code list, based on a review of a representative set of transcripts from both study sites. The code list was informed both by thematic areas arising from the data, as well as through the structure of the IDI guide, which influenced the topics of discussion -using both inductive and deductive coding strategies. This code list was reviewed by a USA team member (NAM) to ensure that it was also representative of USA study data.

The initial code list was tested on additional transcripts (by CM, TSH, BLD and MNM), and any differences and similarities in interpretation were discussed and the code list was revised. If there were any queries related to the USA transcripts, the USA team were consulted. Once agreement was reached, transcripts and the code list were imported into qualitative data analysis software, NVivo version 15 (QSR International), and all transcripts were double coded by two of the four coders (CM, TSH, BLD and MNM) to facilitate reliability. Team members met regularly to discuss and compare coding, and to ensure that inter-coder agreements were met throughout the coding process. At these meetings, code assignments were reviewed, discussed and any discrepancies were resolved by discussing and refining code definitions where necessary. Once all data were coded, coding reports were generated to create summaries of key codes, and data were organised for a thematic analysis. Both USA and SA teams were involved in data interpretation and analysis. Thematic areas are described in the results section below.

Data triangulation was used to compare findings from the VIBRANT study baseline behavioural questionnaire with IDI findings. Participant reports of previous BV diagnosis, treatment for BV and/or vaginal symptoms and history of STIs were compared across IDI and baseline behavioural data.

### Ethical considerations

This qualitative sub-study was approved by the Biomedical Research Ethics Committee (BREC) of the University of KwaZulu-Natal (BREC/00006221/2023) and the Mass General Brigham (MGB) Institutional Review Board (IRB) (2023P003545). All participants provided written or verbal informed consent to participate in the IDI, with separate consent for audio recording of their IDIs. Consenting was conducted in *IsiZulu* or English, depending on participant preference. The study was conducted in accordance with the Declaration of Helsinki (https://www.wma.net/policies-post/wma-declaration-of-helsinki/) – whereby the health, well-being and rights of participants were upheld within the context of ethical research.

The VIBRANT study received separate ethics approval from BREC (Reference: BREC/5620/2023) and the MGB IRB (2023P001035).

## Results

Ninety-six participants were randomised and enrolled in the VIBRANT study (24 at Boston, USA and 72 at Vulindlela, South Africa), and 37 of these participated in the qualitative research (14 from Boston, and 23 from Vulindlela).

At the Vulindlela site, 20 clinical trial participants participated in two serial IDIs, and 3 completed once-off, cases of interest, IDIs. These cases of interest included one participant who did not use her first study product, one participant who shared her study product with her sister, and one who had challenges with study product insertion. There were no refusals to participate in the serial IDIs in South Africa, but there were 2 participants who refused or were unavailable to participate in cases of interest IDIs.

At the Boston site, 12 clinical trial participants completed the first serial IDI and, of these, 10 completed the second serial IDI. One participant did not return for her second IDI, and another was interviewed late for her first IDI, so did not complete a second IDI due to being out of window. The enrolment rate in the VIBRANT study was slow at this site, resulting in a smaller number of enrolled participants. Therefore, recruitment for the IDIs was impacted and, due to a smaller pool of clinical trial participants, a decision was made to reduce the target serial IDI enrolment to at least 10 participants. There were 2 once-off, cases of interest IDIs at this site – one product discontinuer, and one participant who had enrolled but chose not to use the study product. Similarly, due to the smaller number of enrolled VIBRANT study participants, no additional cases of interest were identified. There were no refusals to participate in any IDIs.

The median age of IDI participants was 28 years (26 years at Vulindlela, and 31 years at Boston). Of the IDI participants, 38% (*n* = 14) reported that they had previously been diagnosed with BV and had received BV treatment a median (IQR) of 3 (1-9) – times in the past year (Table 1). The most commonly reported antibiotics used for treatment of past BV and/or vaginal symptoms were metronidazole, clindamycin and azithromycin, but 46% (*n* = 17) reported no antibiotic use. Use of other treatments, most commonly vaginal cream, oral probiotics and boric acid, was also reported. Compared to Boston, participants from Vulindlela had lower prevalence of BV diagnosis (86% versus 9%), antibiotic use (79% versus 22%) and other product use (71% versus 26%). Overall, 19% (*n* = 7) reported having a previous STI diagnosis.

**Table 1:** BV and STI history and treatment

		Vulindlela (*N* = 23)	Boston (*N* = 14)
Previously diagnosed with BV, % (*n*)	Yes	8.7(2)	85.7(12)
Number of times treated in the past year for BV (among those previously diagnosed with BV), median (IQR)		1(1–1)	3(2–9.5.5)
Number of times treated for vaginal symptoms in the past year (among those not previously diagnosed with BV), median (IQR)		0(0–1)	0.5(0–1)
Previous treatment for vaginal symptoms/BV			
Antibiotics used, % *n*	Metronidazole	17.4(4)	71.4(10)
	Clindamycin	0(0)	28.6(4)
	Tinidazole	0(0)	7.1(1)
	Azithromycin	13(3)	7.1(1)
	Doxycycline	4.3(1)	14.3(2)
	Antibiotic injection	13(3)	0(0)
	Do not know	17.4(4)	0(0)
	No antibiotic used	60.9(14)	21.4(3)
Other treatments used, % (*n*)	Vaginal cream	21.7(5)	50(7)
	Home remedies	4.3(1)	7.1(1)
	Oral probiotics	0(0)	28.6(4)
	Vaginal probiotics	4.3(1)	7.1(1)
	Boric acid	0(0)	35.7(5)
	Other treatment	0(0)	7.1(1)
	No other treatment	73.9(17)	28.6(4)
History of STIs (ever diagnosed), % (*n*)	Gonorrhoea	4.3(1)	14.3(2)
	Chlamydia	0(0)	35.7(5)
	Trichomonas	0(0)	7.1(1)
	Herpes	0(0)	7.1(1)
	HPV	0(0)	7.1(1)
	None of the above	52.2(12)	50(7)
	Don’t know if I had STI	43.5(10)	7.1(1)

### Thematic results

Study findings have been thematically organised according to five key themes: (1) prior BV and other vaginal health experiences; (2) previous vaginal health seeking behaviour and treatments used; (3) BV knowledge, attitudes and reactions to BV diagnosis; (4) behavioural changes because of BV; and (5) BV diagnosis disclosure patterns. Any differences and similarities between sites are highlighted.

#### Prior BV and other vaginal health experiences

During the IDIs, none of the participants from Vulindlela specifically reported that they had previously been diagnosed with BV (although two did report previous diagnoses during their clinical trial baseline behavioural assessment). However, almost all Vulindlela IDI participants described previous vaginal health concerns – the majority reporting having had experienced a smelly discharge, itchiness or pain during urination, some of which had been diagnosed and treated as STIs.

*The problem is that itches deep inside where I can’t scratch and where I am unable to touch*,* and if I ever move the flesh*,* I swell up. (Vulindlela*,* 36 years)*

*It is that my vagina would times have a bad odour. Water [discharge from the vagina] would come out that smells. (Vulindlela*,* 24 years)*

Conversely, most IDI participants from Boston reported a previous BV diagnosis, both in their interviews and in the baseline behavioural data. Those who didn’t, made reference to having had BV-related symptoms in the past. Almost all of these participants had experienced multiple recurrences of BV infection, after various treatment options, and were desperate to treat their BV symptoms.

*I had the BV*,* and ever since July*,* I swear to God*,* it’s been BV*,* yeast*,* BV*,* yeast*,* BV*,* yeast—back to back to back to back. (Boston*,* 26 years)*

*After they did the treatment*,* they did another test*,* and the test showed that everything is good. Your vagina is happy*,* but like a month later*,* the symptoms came back*,* did another test*,* and it says*,* “Yes*,* your vagina is now mad.” (Boston*,* 40 years)*.

In addition, participants from Boston also reported having experienced other vaginal/sexual and reproductive health (SRH) concerns. The majority reported experiencing yeast infections or urinary tract infections (UTI). Endometriosis, polyps in the uterus, and an ovarian cyst were also listed as previous sexual health concerns.

*I would be prone to yeast infections or UTIs growing up*,* but I never was officially diagnosed with BV until actually having an IUD [intra-uterine device]. (Boston*,* 24 years)*

*I’ve had a polyp*,* […] recently in the uterus*,* taken out*,* and I’ve had […] a cyst in the ovaries before. (Boston*,* 38 years)*

#### Previous vaginal health seeking behaviour and treatments used

Almost all participants from both Boston and Vulindlela had sought treatment for previous vaginal health concerns. Many of them described recurrence of symptoms, and repeated treatments required. This is further supported by the baseline behavioural data which indicate various types of treatment had been used by participants for vaginal symptoms in the past.

The majority of participants at Vulindlela had been to the local clinic or a doctor for treatment of vaginal discharge, malodour or itchiness, and had been treated with oral tablets and/or an injection. A few had been provided with vaginal cream to insert and/or apply, and only two described being given a vaginal tablet to insert. Participants did not provide details of the names of the treatment they had used.

*It would come back at a later stage, this discharge.* *[….]* *I would go to the clinic […] [t]hey would give me pills and something else...Ya, last time, […] they gave me pills and injected me. [….] [* *I: Oh, the cream?]* *No, they have never given me. […] It was drinkable pills then an injection. (Vulindlela, 36 years)*

*[W]hen I had a discomfort*,* and it was severe. It made me go and see the doctor. [….] Because she gave me a…was it a cream? A vaginal cream*,* that I inserted at night. It was cream only. (Vulindlela*,* 34 years)*

*There was… [a tablet that you had to insert in your vagina] [….] It is shaped exactly like the tablet that from here [research site] and its colour. [….] They [local clinic staff] gave me the one that you insert. [….] It helped me because what I had got treated*,* I didn’t have it anymore. (Vulindlela*,* 24 years)*

Some participants from Vulindlela described challenges accessing treatment/care from local clinics, largely due to staff attitudes, and preferred to access treatment elsewhere, for example private pharmacies.

*It’s just that some nurses have tendencies that are not alright. When you come to a nurse because you have a problem*,* but they will call another one*,* you see now? “Tell us again*,* what is wrong with your vagina?” You see*,* now you are getting embarrassed*,* [….] You leave the clinic not feeling alright. So*,* the pharmacy is better. The pharmacy doesn’t ask anything. You get there*,* you say the problem that you are facing*,* they get you what will help you and you leave. (Vulindlela*,* 25 years)*

Most participants from Boston reported previously being prescribed antibiotics, such as metronidazole, for their BV symptoms. Some reported taking oral antibiotics, others reported using a cream or suppositories for treatment. Many of them also described using boric acid to relieve their symptoms. Treatment success was reportedly not high, with many participants reporting using multiple treatment options for recurrent symptoms.

*I have been trying too many options. The doctor*,* she just send[s] more antibiotics*,* more antibiotics*,* more*,* more*,* and then nothing is being helpful. [….] I have been using the boric acid [….] That was working. [….] At least feel better. Yeah*,* the discharge minimized*,* and that was helpful. (Boston*,* 38 years)*

*I feel like in the past when I’ve used the metronidazole cream*,* I had very minimal relief*,* and same with the [vaginal] tablet too. Maybe I’ve grown a tolerance to both*,* I don’t know*,* but I feel like the tablet— it was definitely more effective than both. Upon my initial diagnosis*,* I was prescribed metronidazole tablets*,* and with that*,* my symptoms did not change at all. (Boston*,* 24 years)*

The majority of participants from Boston reported being prescribed medication by their doctors, gynaecologists or primary care physicians.

*Everything that I know about my vagina is questions I’ve asked my gynaecologist*,* and I just trust whatever—whatever she says*,* I trust. (Boston*,* 26 years)*

##### Self-care practices

There were also various reports of self-care/self-help practices used by participants from Vulindlela and Boston to treat or alleviate their symptoms – both in IDI and baseline behavioural data. Some Vulindlela participants described steaming or hygiene practices.

*People just know that if you have a problem there [vagina]*,* you steam using water and onion*,* done! (Vulindlela*,* 25 years)*

*I would just take water*,* pour it in the basin and bath to make it [her vagina] cooler because it was painful and inflamed*,* and when I have washed it with water the pain would decrease*,* but there is no pill that I can say helped me*,* no there isn’t. (Vulindlela*,* 36 years)*

A couple of participants from Vulindlela reported using citro soda/potassium citrate to alleviate their vaginal malodour, and there were reports of searching online or via social media for remedies.

*I used to buy citro soda […] I used to drink that thing. […] I learned on TikTok [social media platform] that it resolves smelly odour*,* which is what I had*,* so I used to drink it and it would help me for like two days and will have to do it again and again. (Vulindlela*,* 20 years)*

*I talk to my friend*,* the two of us because when she has a problem*,* she asks me and says*,* “you know*,* I am going through such and such”. We usually sit down*,* and search on google on what could help us. (Vulindlela*,* 27 years)*

A few participants from Boston described scepticism of the healthcare system which led to self-help treatment. Some bought over-the-counter treatment, with some reporting using probiotics and one reporting using cannabidiol (CBD) suppositories for BV treatment. In Boston, one participant mentioned that some people would use yoghurt, steam or douche their vaginas as an alternative to accessing the health system.

*I think people have fraught relationships with the medical system […]*,* I think that’s part of the reason why many women have turned to these alternative products that might be harmful*,* but they’re desperate for anything and they don’t feel like they can get the help they can within the medical system*,* unfortunately. People who put yoghurt in their vaginas or use steam douching or things like that. (Boston*,* 25 years)*

*In the past*,* I’ve also used CBD repositories. Especially while I was on my period. I heard that that would relax my muscles somehow. (Boston*,* 24 years)*

#### BV knowledge, attitudes and reactions to BV diagnosis

No participants from Vulindlela described any knowledge about BV prior to joining the VIBRANT study. Once they joined, they learned about it and demonstrated varying degrees of understanding of BV. All participants from Boston demonstrated that they understood/had some knowledge about BV.

*I know that BV is found in almost 25% of the women worldwide. You can see it when you have a white discharge that has a bad odour. It happens sometimes that the vagina might be itchy or have pimples or experience inflammation. […] I didn’t know all that knowledge [before joining the study]. I got it when I was in the study here. (Vulindlela*,* 19 years)*

Participants were asked to describe how finding out about their BV status impacted them in their daily lives. This was closely related to their attitudes to BV. The majority of participants from Vulindlela had not previously been diagnosed with BV and were first diagnosed during participation in the VIBRANT study. Of these, the majority first expressed fear, shock or surprise about their diagnosis. However, this was soon replaced with relief that their BV could be treated during the study.

*It scared me*,* I don’t want to lie. I got scared*,* and I was very shocked that […] I didn’t think that something like this could be found on me. […] I thought that it was a bad thing*,* yho! I thought it was just a bad thing*,* shame*,* and maybe something big*,* maybe that cannot be cured. […] I learnt about it and they said that it is curable. (Vulindlela*,* 25 years)*

A couple of Vulindlela participants had previously suspected that they had a vaginal health issue and were happy to be diagnosed. Others had no concerns with their diagnosis, although were happy to know about their health status.

*[W]hen I got here and it [BV] was explained do me*,* by knowing myself as a woman*,* I had already seen that there is an infection that I have. (Vulindlela*,* 34 years)*

In contrast, the majority of participants from Boston were aware of their BV status before joining the study. They described how the knowledge of having BV was not as impactful as actually experiencing BV. For them, BV was viewed as negative – described as irritating and uncomfortable, impacting daily life, including sex, intimacy and social situations.

*Knowing that I have BV is a non-issue. It’s the BV itself which is the issue. [….] I can’t have sex easily. When I do have sex*,* it hurts. Not to mention that just walking around all day—I have all this constant discharge. It’s painful*,* it’s burning. It dries up my everything down there*,* and it smells like hell. The thing is*,* I take a shower every day. I’m a clean person. I changed my underwear. I’ve always been a clean person. Now that I have this recurring BV*,* it makes me feel dirty*,* because nothing that I do works*,* and it always smells bad. I can’t have sex*,* and I hate that. I can have sex*,* but it hurts like hell. (Boston*,* 26 years)*

*[BV] makes you feel weird. I guess it’s something you can smell*,* but nobody else can smell*,* but you think everybody else can smell it. The fishy odour is nasty. […] Especially being a female. You’ll be sitting around a group of people*,* and you’re like*,* “I smell myself. I’m pretty sure everyone else smells it”. (Boston*,* 38 years)*

Some Boston participants described that they felt better knowing that there were other people who also had BV and that they were not alone. This had positive implications for their wellbeing.

*My views [on BV] haven’t changed. What I have learned is*,* again*,* I’m not the only one*,* so don’t feel ashamed or embarrassed. (Boston*,* 34 years)*

##### Vaginal health and hygiene practice

Some participants at both sites described their vaginal cleaning practices, especially to address their BV symptoms. Some participants from both Boston and Vulindlela made use of feminine washes and/or wipes to address malodour and discharge that were experienced from BV.

*[Friends] usually ask me*,* “hey*,* what makes you not to have a smell?” Then I told them that*,* “as much as I use GynaGuard [intimate wash soap] when I bath”. I told them they must use it. (Vulindlela*,* 27 years)*

*I’ll use feminine washes. Feminine washes*,* detox pearls*,* boric acid. That’s as far as it goes. (Boston*,* 28 years)*

Other participants from both sites described avoiding use of soap or using gentle soap to clean their vaginas.

*I try to avoid getting soap anywhere near the inside. I try to keep it as far from the outside as I can*,* or on the outside. (Boston*,* 24 years)*

Frequency of washing varied between individuals, from 1 to 3 or 4 times per day.

*I usually washed in the morning*,* and I would wash again when I was going to bed. (Vulindlela*,* 27 years)*

*I shower in the morning*,* and I shower after the gym*,* and then sometimes I shower at night*,* depending if I go out. (Boston*,* 40 years)*

A few Vulindlela participants reported changes in their hygiene practices after their BV diagnosis during the study.

*[B]efore I came*,* when I had the*,* the discharge that was coming out with a smell*,* I ended up trying to buy wipes*,* you see? […] But when I got here [research centre]*,* we were told*,* “For now*,* can you stop using soap”. I would then just bath my body then take something to get water and clean it with cold water. (Vulindlela*,* 34 years)*

*They taught me that as I still have BV*,* [….] when I was cleaning the private part*,* I must not insert a finger*,* because the finger has germs. (Vulindlela*,* 24 years)*

Similar to Vulindlela, some Boston participants changed their vaginal hygiene practices during the study, including showering/cleaning more regularly.

*As far as instead of two showers a day*,* it would be three showers a day type thing. (Boston*,* 33 years)*

##### Changing clothes/laundry practices

In addition, Boston participants reported that they had to change their underwear more regularly or do laundry more often because of the associated discharge and odour of BV.

*It comes with strong odour*,* discharge. Uncomfortable*,* I have to change my underwear twice to three times a day sometimes. (Boston*,* 32 years)*

##### Changes to sexual behaviour/practices

Changes to sexual practices as a result of BV diagnosis were not discussed at Vulindlela. This is possibly because participants were first diagnosed with BV during the study, therefore there was no previous real life experience to draw on. However, one participant reported that because of her BV, she was advised to use a condom during sex.

*They taught me that as I still have BV*,* I have to use a condom when I have sex. (Vulindlela*,* 24 years)*

Participants from Boston reported more changes in sexual behaviour as a result of BV – some reported abstaining, and others reported reducing the amount of sex or changing sex acts (e.g. no more oral sex). These changes were made because of being self-conscious about the BV related malodour, because of discomfort during sex, and their perceived impact of sex on the recurrence of BV symptoms.

*[T]he symptoms are very––it affects your life a lot. I’m not gonna have sex with someone when it smells bad. Yeah*,* it makes me very uncomfortable*,* very self-conscious. Yeah*,* it made me feel kind of gross. (Boston*,* 40 years)*

*Oral [sex] had already been impossible ‘cause I’d been having recurrent BV*,* and that recurrent like smell and discharge. (Boston*,* 26 years)*

#### BV diagnosis disclosure patterns

Only some participants from Vulindlela and Boston disclosed their BV status to others. Participants from Vulindlela were more likely to disclose to female family members (mothers, sisters, grandmothers and/or aunts), whereas participants from Boston were more likely to disclose to their partners. Although, there were a few participants from Vulindlela who did disclose their BV diagnosis to their partners, and a few from Boston who did tell female family members about their BV. The level of detail disclosed also varied between participants.

*I just thought*,* okay*,* I will tell my mother*,* even though I will not tell her every single thing*,* but I will tell her that there is this kind of a pill*,* and they say that it treats the BV disease. Even though I will not tell her deeply about what it is. (Vulindlela*,* 19 years)*

*[S]o my partner’s actually in medical school*,* so actually it’s helpful. I think with my partner*,* I’m pretty much completely transparent about all aspects of it [BV]. With my mom*,* I think I was—maybe I didn’t need to be this honest*,* but I basically told her the reason*,* I was like*,* “I think this happened is because I had sex without a condom.” She was like*,* “Oh my god.” (Boston*,* 25 years)*

A few participants also disclosed to friends. Some participants reportedly disclosed their BV status for altruistic reasons, as they wanted others to get information and help for BV if necessary.

*I used to tell others too because some people don’t know about the illness called BV*,* which comes out of a person’s private part*,* of a woman. (Vulindlela*,* 20 years)*

*I feel like dealing with this condition since the end of last year has very clearly impacted my life*,* and so I feel like if I’m catching up with a friend and they’re like*,* “How are you doing?” I’m like*,* “Well*,* I’ve been dealing with some health stuff.” Then very naturally*,* they’re like*,* “Oh*,* what kind of health stuff?” I’m like*,* “Okay*,* well there’s this thing called BV.” […] I’ve tried to be open about it because I think part of the reason why conditions like this aren’t studied is because people are hush hush about it because it is extremely embarrassing and stigmatizing*,* but I think openness is probably better than not openness. (Boston*,* 25 years)*

Participants had varying reasons for disclosing to some and not to other people, as described by one Vulindlela participant:

*It was only my Mother, it was my Mother and my sister who knew. [….] I didn’t tell my partner.* *[I: [F]riends, have you ever told your friends?]* *[….] Oh, what made me to tell my friends […], so I thought maybe they could also be able to come here [to the research centre] … to get this research that is about BV. Maybe they can also get helped as I also got helped. What made me not to tell my partner is because he was going to judge me. He likes to be judgemental. (Vulindlela, 25 years)*

Some people did not disclose their BV status at all:

*I don’t share with people. Not too much because it’s a study product*,* but because it’s bacterial vaginosis. No one wants to talk about that. If it was a study product for any other—if it was for throat pain*,* I would happily share it with the world*,* but no[one] needs to know I have BV. (Boston*,* 40 years)*

## Discussion

Similar to previous research, women in this study had poor levels of awareness of or knowledge about BV prior to being diagnosed with it [3]. In this study, few South African participants had previously been diagnosed with BV, compared with the USA participants, who had mostly had a previous BV diagnosis. As a result, most USA participants demonstrated a good knowledge of BV, whereas South African participants had limited knowledge or awareness before their participation in the VIBRANT study. There is a paucity of literature comparing BV knowledge across these different country contexts – much of the existing qualitative research has been conducted in Australia [8, 12, 18]. Despite varying knowledge, participants from both countries reported similar past SRH and vaginal health experiences – including increased vaginal discharge and malodour, typical of BV [1, 8–10], as well as recurrence of these symptoms after treatment.

In South Africa, the standard treatment guidelines for primary health care recommend that STIs are syndromically managed in order to be able to treat more than one condition that could be occurring at the same time [15]. Syndromic management rather than testing for BV is further exacerbated by limited resources in primary health care settings. This practice of treating symptoms rather than testing for STIs or BV specially, could be the reason for limited previous diagnosis and knowledge in South African participants.

Participants in this qualitative study described various treatment strategies and drug formulations accessed for their vaginal health conditions or BV symptoms, prior to their participation in the VIBRANT study [21]. These included oral tablets, injections, vaginal creams, and vaginal inserts/tablets. Specifically, antibiotics such as metronidazole, were reported by USA participants as recommended for BV treatment [4, 11]. However, attitudes of healthcare workers and reduced faith in treatment because of recurrence of symptoms resulted in participants also reporting trying self-care treatment strategies. This move to alternative, self-help treatment strategies such as boric acid, douching, probiotics and yoghurt has also been reported elsewhere [3, 4]. In South Africa, where there is a strong reliance on traditional healing in some cultures, it is surprising that participants did not describe accessing these types of services. Although there were reports of vaginal steaming and douching by a few South African participants.

In this study, participants who reported previous BV diagnosis described how their BV symptoms, malodour and vaginal discharge, as well as the concern that sex could trigger BV, had impacted their sexual behaviours – including abstinence and reducing frequency of sex. Previous research describes that decreased sexual quality of life can also result in depression, distress and relationship disruptions [10]. Furthermore, participants in this study demonstrated shame and embarrassment as a result of BV symptoms. These negative psychosocial impacts of BV can result in reducing quality of life [4, 8, 11]. However, participants in this study revealed that knowing that others suffered from BV made them feel less ashamed, which had positive implications for their wellbeing.

Experiences of BV also resulted in participants reporting changes in their vaginal hygiene and/or washing practices, both to decrease symptoms as well as to reduce potential for BV recurrence. Initially, participants described more frequent washing, and the use of feminine washes or wipes to address malodour and discharge. However, as highlighted elsewhere, soaps used to wash the vagina have been viewed as triggers for BV [19], and use of soap free washes have been reported for vaginal hygiene [12]. Similarly, some participants in this study described avoiding soap or using gentle soaps to clean their vaginas after BV diagnosis. In addition, USA participants in this study described frequently changing their underwear, or more frequently washing their clothes because of the increased vaginal discharge and malodour of BV. Women in other research also report changing their underwear more frequently [12], or choosing clothing accordingly – choosing cotton underwear, avoiding tight clothing, and wearing clothing that encourages ventilation to manage their BV symptoms [11, 12].

South African participants were more likely to disclose their BV diagnosis to female family members and USA participants were more likely to disclose to male sexual partners. South African participants often lived with other female family members, which could have impacted on their decisions to tell them. Although USA participants did not always live with their male partners, many were in committed relationships. Other studies have demonstrated that disclosure to certain people is often related to the need for encouragement and support [8, 11]. Some participants from both sites disclosed to friends for altruistic reasons – they wanted to provide information and guidance to others who may also experience BV. Previous research found that single women were less likely to discuss BV diagnosis with casual partners and more likely to discuss it with friends, whereas women in relationships were more likely to discuss their concerns with regular partners, but also commonly discussed their diagnosis with friends as a means of information gathering [8]. By disclosing to partners, women have been able to get support with managing symptoms in intimate exchange/sexual encounters [11].

Previous research [8] has demonstrated how a theoretical framework, such as social constructionism, can be used to describe how “individual’s perceptions of reality and the meanings they give to phenomena are shaped by the social and cultural norms” (p.2) within their context. In this study, participants have similar experiences of stigma and shame related to genital symptoms, especially in SA. This impacts on participants’ experiences of BV and their attitudes towards health-seeking behaviour, which in turn affects knowledge of BV.

### Future recommendations

This research highlights the lack of adequate diagnosis and treatment of BV in the healthcare setting, especially in the South African population. There is a need for healthcare staff to learn to differentiate BV from other conditions, and to treat it appropriately [11]. Healthcare providers may need further training on recognising BV related symptoms, to facilitate screening, testing and treatment of BV. However, in resource constrained settings, such as South Africa, where syndromic management of STIs is recommended [15] there may be a need for further research to justify changes in guidelines. In spite of various clinical trials which have looked at interventions to prevent recurrent BV after antibiotic treatment [24], some of which are ongoing (NCT06933420 and NCT064469164), there is a need for further studies to identify possible strategies to treat BV so that there is minimal recurrence [21].

Healthcare providers have an obligation to provide clients with better BV related information, knowledge and support through counselling [10, 12]. Furthermore, they should counsel women to identify possible BV related symptoms so that they actively seek out appropriate screening and testing for treatment. The benefits of medical versus self-care treatment strategies need to be understood, and awareness and education should also target this. Education and support need to extend beyond the healthcare setting, and there is a need to create reliable and trustworthy sources of information for the public. Targeted advertising campaigns and education in schools and communities around SRH and BV should be developed [12], to create increased awareness of BV and BV symptoms. With access to improved, reliable public knowledge, misinformation can be corrected and stigma and psychosocial impacts of BV can be better managed and supported.

In order to assess the burden of BV, more quantitative studies can be conducted to explore the impact of/association of BV with emotional, sexual and social health [10]. Through this, targeted support can be provided to people who are diagnosed with BV. Appropriate diagnosis and treatment and support are critical to ensure that BV is adequately managed and that quality of life and associated stigma and health risks of BV are eliminated.

### Limitations

As with any study, there are some limitations. Due to the qualitative nature of the study, the sample size is relatively small and therefore the findings are limited in generalisability, but are exploratory in nature. Furthermore, participants in this study may not be representative of the broader population as they were enrolled in a clinical trial, and may have had different health seeking behaviours from the general population. Therefore, findings should be interpreted in this context of clinical trial participation. Although there are some differences between the USA and South African participants’ reports, these may be specific to the regions in which the study was conducted. However, there are some findings which are more general, and which highlight some of the important challenges with BV knowledge and treatment experiences, which are useful for future research and education on BV.

## Conclusions

BV knowledge, attitudes and experiences are related to previous diagnosis and treatment. However, experiences of symptoms impact individuals’ behaviours, regardless of previous diagnosis. Experiences of discharge, malodour and discomfort all have psychosocial impacts which cannot be appropriately addressed without access to appropriate counselling and treatment options. There is a need to ensure that there is ongoing research for effective BV prevention and treatment options that can be made available and accessible to women with BV globally.

## Supplementary Information

Supplementary Material 1.

Supplementary Material 2.

Supplementary Material 3.

Supplementary Material 4.

Supplementary Material 5.

## Data Availability

Access to the data from this sub-study may be requested through submission of a research concept to the corresponding author at [cecilia.milford@gmail.com](mailto: cecilia.milford@gmail.com). The concept must include the research question, data requested, analytic methods, and steps taken to ensure ethical use of the data. Access will be granted if the concept is evaluated to have scientific merit and if sufficient data protections are in place.

## Abbreviations

BREC: Biomedical Research Ethics Committee
BV: Bacterial vaginosis
CBD: Cannabidiol
HIV: Human immunodeficiency virus
IDI: In-depth interview
IQR: Interquartile range
IRB: Institutional Review Board
IUD: Intra-uterine device
LBP: Live biotherapeutic product
MGB: Mass General Brigham
SA: South Africa
SRH: Sexual and reproductive health
STI: Sexually transmitted infection
USA: United States of America
UTI: Urinary tract infection
VIBRANT: Vaginal lIve Biotherapeutic RANdomized Trial
