# Supplementary material for: Bacterial Vaginosis [BV] knowledge, attitudes and behavioural changes after BV diagnosis among women enrolled in a clinical trial in Boston, USA and Vulindlela, South Africa: a qualitative study
Source: BMC Womens Health. 2025 Dec 12;26:32. doi: 10.1186/s12905-025-04210-3 (PMC12817389; doi:10.1186/s12905-025-04210-3)
Supplement: Supplementary file 1 — Supplementary Material 1. [file 12905_2025_4210_MOESM1_ESM.pdf]

User experience and preferences of a vaginally delivered live biotherapeutic product in South African women participating in the VMRC 001 study

Special Case IDIs: Other

PARTICIPANT ID NUMBER	DATE (DD/MMM/YY)	START TIME	END TIME	INTERVIEWER INITIALS

[*Start Recorder and Read Introduction*]: **I have started recording now. The participant ID is ____, the date and time is _____. My name is _________________. Thank you again for your willingness to be in this study. The main goal of this discussion is to better understand your experience participating in this study and using the study product. I want to remind you that there are no right or wrong answers, and what we discuss here will be kept confidential; we will not share your personal information or responses with anyone outside of the study.**

If during our discussion, there are issues or concerns that you would like to talk about, feel free to bring them up; I will take note of them and answer them directly after the interview. If I cannot answer them, I can refer you to someone who may be able to help.

**Section 1: Background**		
1.1	Can you tell me a little bit about yourself?	• Can you tell me about your background, work, family and social life?<br>• Who is generally responsible for making sexual health decisions in your family and community?<br>• How well known are vaginal products in your community?<br>• Can you tell me about attitudes people in your community have to vaginal products?
1.2	What do you know about bacterial vaginosis (BV)?	• What did you know about this before joining the study?<br>• How has finding out about your BV diagnosis affected you and your daily life?<br>• How have your views on BV changed since joining the study and learning more about it?

1.3	Have you used vaginal products prior to this study?	• What products have you used?<br>• What were your initial thoughts/feelings during the first time you used a vaginal product?<br>• What did you use these products for (for example, products that enhance sex, dry or moisturize the vagina, medical products that are inserted in the vagina, or vaginal products for menses, or HIV prevention products)<br>• Have you been enrolled in any other research studies that were researching any other vaginal products before? If so, what were the studies for? How was your overall experience in these studies?
1.4	Why did you decide to join this study?	• Probe for concerns with vaginal health<br>• Probe about other health assessments, reimbursement, etc.
**Section 2: General Vaginal Tablet Insertion and Use**<br>*We are now going to talk about your experiences in the study. Please feel free to tell us about what you liked and disliked. Your opinions are important.*		
2.1	Tell me what you thought of the vaginal tablet when you first saw it and heard about how it is used.	• Size, shape, colour?<br>• How would others in your community feel about it? Your friends? Your family?
2.2	How did you feel about the way you had to insert the vaginal tablet into your vagina?	• What did you use to insert it? Describe the insertion process.<br>• Describe your experience using the applicator for insertion – do you think it made insertion easier or more difficult? How would you compare this with the choice of using your fingers for inserting the vaginal tablet?<br>• Please describe any challenges and successes encountered.

2.3	How did you feel about inserting the product into your vagina on your own?	Explore:<br>• Comfort with vaginal tablet insertion.<br>• Familiarity with your vagina.<br>• Confidence with placement.<br>• Number of insertions/attempts when inserting for the very first time.<br>• Overall, how difficult or easy was the insertion process the first time you tried it?<br>• Did you find it easier the second time and so forth?<br>• Was there any change in your experience with applicator use over time? Explore.
2.4	Can you describe your experience using the vaginal tablet daily for 7 days?	• Explore comfort with use.<br>• What side effects did you experience from the vaginal tablets? Any discharge?<br>• What did you do about the side effects?<br>• Did it get easier to use over time?<br>• What if anything would you change about the tablet (size, shape, colour, frequency of use)?<br>• Compare the use of the vaginal tablet vs other vaginal products.<br>• Did you wash your vagina more or less during product use? Explore.
2.5	Some people find it difficult to remember to take medication daily. Describe your daily routine for inserting the vaginal tablet.	• Did you ever forget to use it? Explore reasons and what they did if they forgot it.<br>• How did you remember to insert the vaginal tablet?<br>• What time of day did you insert the tablet? Why that time specifically?<br>• Did anyone else remind you to insert it? Who?
2.6	What do you think are the pros and cons of using a vaginal tablet to treat BV?	• Is there anything that would make you more likely to use it?<br>• Is there anything that would make you less likely to use it?<br>• How would you rate the experience overall (e.g., positive, negative, or neutral)?
2.7	*Questions specific to this participant's circumstances:*<br>How did your experiences around the event/experience (e.g., discharge) affect your product use?	• How concerned were you when the event/experience occurred?<br>• How did you respond?<br>• Have you informed clinical trial staff about the event/experience?<br>• How do you view the study product now?
**Section 3: Vaginal Tablet Discreetness and Disclosure**		

I would now like to ask you questions about your decisions regarding disclosing your use of the vaginal tablet and discreetness use of the vaginal tablet.		
3.1	Using the vaginal tablet is a personal choice, and some people may prefer to keep this private until they are ready to talk about it, while some would prefer to disclose that they are using a vaginal tablet much earlier than others. How did you decide who you did and didn't tell about using the vaginal tablet?	• Who did you disclose to? (family, friends, partner/s?)<br>• If they did disclose to someone: How did you feel leading up to the disclosure (were you nervous/scared, calm, or excited)?<br>• What did you tell them (those that you disclosed to)?<br>• How did they respond to your disclosure? Explore for all disclosed to.<br>• Why did you disclose?<br>• If chose not to disclose, why did you choose not to disclose?
3.2	Did anyone help you to remember to insert your vaginal tablet during the course of the study?	• Who? How did they assist?
3.3	How important was it for you that you could use the vaginal tablet discreetly, i.e., in secret?	• Describe any times where someone found out about your vaginal tablet use without you telling them? What happened? How did they respond?
**Section 4: Life Fit and Relationship Fit**<br>*Now I am going to ask you questions about how the vaginal tablet impacted your life in general as well as your sexual relationship with your partner. Please remember that whatever you tell me will remain confidential, so please feel free with sharing your responses.*		
4.1	Can you tell me more about your current sexual partner(s)?	• 1 main partner or more? How long been together? How would you describe your relationship(s)?<br>• Please describe any experience of vaginal sex during the 7 days of product insertion? Did it improve sex? Make it worse? How?<br>• We know that sex is sometimes spontaneous, and that you may not always have been able to wait 6 hours after product insertion before sex – please tell us if this happened to you, and how sex was during this time?<br>• Do you think your peers/community members would be able to delay sex after product use?<br>• Did your partner/s know about the vaginal tablet? (If not described above, explore disclosure to partners, e.g., how did they respond?)<br>• Did your partner/s notice anything different during sex? Explore – what did they say about it?

4.2	Please can you tell me how the vaginal tablet fitted or did not fit into your life and in your relationship?	• Where did you keep the tablets? Why did you keep them there and how did you decide?<br>• What adjustments did you have to make to your life when using it?<br>• Did it have any impact on how you engaged with others? With your partner?<br>• Were you nervous that other people would find out?
4.3	How influential were your partner, your friends and family, and others in the community on vaginal tablet use?	• Who had an influence and what was it like?<br>• How did they change your attitudes and behaviours?<br>• Did anyone support you in any way other than reminding you to insert? If so, how (e.g., driving her to clinic visits, checking in on how she's doing)?<br>• Explore any myths related to the vaginal tablet.<br>• Explore any beliefs (religious/traditional) related to the vaginal tablet.
**Section 5: Study Participation**		
5.1	How did you feel about collecting vaginal swabs at home for this study?	• Explore challenges, facilitators – was it easy or hard to do?<br>• Where did you store the vaginal swabs? Why did you choose that place?
5.2	How did you feel about taking the antibiotics?	• Did you experience any challenges with this?<br>• How easy or difficult was it to adhere and complete the antibiotic course? Did you miss any doses?<br>• Any side effects?
5.3	How are you finding the daily diary?	• How easy or difficult is it to keep up with filling in the diary?<br>• How helpful are you finding the diary to be?
5.4	How have your interactions with study staff been throughout the study?	• What kinds of information have they provided about the study? Study product/vaginal tablet?<br>• How comfortable are you telling them about problems you may have experienced? Elaborate.
5.5	Did you see or talk to any friends or other women while at the research site?	• What kinds of experiences did they describe about the trial?
**Section 6: Future Use**<br>*Now we are going to talk about factors that would make women want to use the vaginal tablet in the future.*		

6.1	How do you think women in your community would feel about using a vaginal tablet like this to treat BV?	• What concerns or questions do you think women in your community would have?<br>• Discreetness.<br>• Design.<br>• Number of insertions per certain timeframe.<br>• What would they find challenging?
6.2	How would you feel about using the vaginal tablet for treatment of BV in future?	
**Section 7: Conclusion**		
7.1	Now that I have asked you all the questions that I wanted to ask you, I might have left out very important things that we should know about the vaginal tablet. Is there anything else that I may have missed that you would like to tell me about your experiences with the vaginal tablet?	

Thank you for your input and time. We appreciate what we have learnt from you. The end time of interview is_____________ [Turn off recorder]
