# Supplementary material for: Bacterial Vaginosis [BV] knowledge, attitudes and behavioural changes after BV diagnosis among women enrolled in a clinical trial in Boston, USA and Vulindlela, South Africa: a qualitative study
Source: BMC Womens Health. 2025 Dec 12;26:32. doi: 10.1186/s12905-025-04210-3 (PMC12817389; doi:10.1186/s12905-025-04210-3)
Supplement: Supplementary file 3 — Supplementary Material 3. [file 12905_2025_4210_MOESM3_ESM.pdf]

User experience and preferences of a vaginally delivered live biotherapeutic product in South African women participating in the VMRC 001 study

Special Case IDIs: People who choose not to participate

PARTICIPANT ID NUMBER	DATE (DD/MMM/YY)	START TIME	END TIME	INTERVIEWER INITIALS

[*Start Recorder and Read Introduction*]: **I have started recording now. The participant ID is ____, the date and time is _____. My name is _________________. Thank you again for your willingness to be in this interview. The main goal of this discussion is to better understand your decision on not participating in this study or using the study product. I want to remind you that there are no right or wrong answers, and what we discuss here will be kept confidential; we will not share your personal information or responses with anyone outside of the study.**

If during our discussion, there are issues or concerns that you would like to talk about, feel free to bring them up; I will take note of them and answer them directly after the interview. If I cannot answer them, I can refer you to someone who may be able to help.

**Section 1: Background**		
1.1	Can you tell me a little bit about yourself?	• Can you tell me about your background, work, family and social life?<br>• Who is generally responsible for making sexual health decisions in your family and community?<br>• How well known are vaginal products in your community?<br>• Can you tell me about attitudes people in your community have to vaginal products?
1.2	What do you know about bacterial vaginosis (BV)?	• What did you know about this before joining the study?<br>• How has finding out about your BV diagnosis affected you and your daily life?<br>• How have your views on BV changed since learning more about it?

1.3	Have you used vaginal products prior to this study?	• What products have you used?<br>• What were your initial thoughts/feelings during the first time you used a vaginal product?<br>• What did you use these products for (for example, products that enhance sex, dry or moisturize the vagina, medical products that are inserted in the vagina, or vaginal products for menses, or HIV prevention products)?<br>• Have you been enrolled in any other research studies that were researching any other vaginal products before? If so, what were the studies for? How was your overall experience in these studies?
1.4	Why did you decide that you did not want to join this study?	• Probe for fears/concerns with vaginal health or with the study product or with the study procedures<br>• Probe about any other feelings on health assessments, reimbursement, etc.
**Section 2: General Vaginal Tablet Insertion and Use**<br>*We are now going to talk about thoughts about the study. Your opinions are important.*		
2.1	Tell me what you thought of the vaginal tablet when you first saw it and heard about how it is used.	• Size, shape, colour?<br>• How would others in your community feel about it? Your friends? Your family?
2.2	How did you feel about the way that you would have had to insert the vaginal tablet into your vagina?	• What were your initial thoughts while receiving the information on the tablet?<br>• Please describe any perceived challenges/experiences that you felt you would have experienced with product insertion?<br>• What do you think about using an applicator versus your fingers for vaginal tablet insertion?
2.3	Can you describe your thoughts about using the vaginal tablet daily for 7 days?	• Thoughts about daily use? How to remember/what they would use as a reminder?<br>• Thoughts about possible side effects?<br>• What, if anything, would you change about the tablet (size, shape, colour, frequency of use)?<br>• Compare the use of the vaginal tablet vs other vaginal products (if applicable).

2.4	What do you think are the pros and cons of using a vaginal tablet to treat BV?	• Is there anything that would make you more likely to try it in future?<br>• Is there anything that would make you less likely to try it in future?<br>• What changes would you make to the tablet based on your knowledge?
**Section 3: Vaginal Tablet Discreetness and Disclosure**<br>*I would now like to ask you questions about your decisions regarding disclosing your use of the vaginal tablet and discreetness use of the vaginal tablet.*		
3.1	Using the vaginal tablet is a personal choice, and some people may prefer to keep this private until they are ready to talk about it, while some would prefer to disclose that they are using a vaginal tablet much earlier than others. How would you decide who you would and wouldn't tell about using the vaginal tablet?	• Who would you disclose to? (family, friends, partner/s?). Why those people specifically?<br>• How do you think you would feel leading up to disclosing (to family, friends, partner/s)?<br>• How do you expect they would respond to your product use? Explore for all disclosed to.<br>• If they would choose not to disclose, why?
3.2	How would you remember to insert your vaginal tablet?	• Would you ask anyone to assist? Who? Why?
3.3	How important would it be for you that you could use the vaginal tablet discreetly, i.e., in secret?	• Describe
**Section 4: Life Fit and Relationship Fit**<br>*Now I am going to ask you questions about how the vaginal tablet impacted your life in general as well as your sexual relationship with your partner. Please remember that whatever you tell me will remain confidential, so please feel free with sharing your responses.*		
4.1	Can you tell me more about your current sexual partner(s)?	• 1 main partner or more? How long been together? How would you describe your relationship(s)?<br>• Do you think you/your partner would be able to wait 6 hours after product insertion before sex?<br>• Do you think your peers/community members would be able to delay sex for 6 hours after product use?<br>• Do you think product use would impact on how you experience sex?<br>• Do you think it would impact on your partner's experience of sex? What do you think he would say about it?

4.2	Some people find it difficult to remember to take medication daily. Describe whether you think inserting the vaginal tablet would fit into your daily routine if you ever chose to use it.	• Explore reasons she may forget.<br>• Explore ways she may use to remember to insert the vaginal tablet.
**Section 5: Other Study Components**		
5.1	What did you think about the requirement for collecting vaginal swabs at home for this study?	• Explore challenges, facilitators?
5.2	What did you think of the daily diary that you would have had to fill in?	• Did it seem like it would be easy or difficult to fill in? Why?<br>• Do you think the diary would have been helpful for you during the study?
5.3	What did you think about in terms of taking part in study procedures? (e.g., the tests, coming for study visits, returning samples, etc.)	• How did you feel about the various study procedures? (e.g. nervous, calm, neutral).<br>• What impact did you think they would have on your everyday life?
**Section 6: Future Use**<br>*Now we are going to talk about factors that would make women want to use the vaginal tablet in the future.*		
6.1	If the product is found to be effective, what could make you interested in using a vaginal tablet to treat BV in future, if anything?	• Explore product attributes (size, colour), or other products such as oral, injection, etc.<br>• Explore dosing requirements<br>• Discreteness
6.2	How do you think women in your community would feel about using a vaginal tablet like this to treat BV?	• What concerns or questions do you think women in your community would have?<br>• Discreetness.<br>• Design.<br>• Number of insertions per certain timeframe.<br>• What would they find challenging?
6.3	If this vaginal tablet is made available, do you think other people in your community would want to/would use it?	• Why/why not?<br>• Where would you prefer to get it from (e.g. clinic, pharmacy, etc)?<br>• How much would they pay for it if necessary?

**Section 7: Conclusion**	
7.1	Now that I have asked you all the questions that I wanted to ask you, I might have left out very important things that we should know about the vaginal tablet. Is there anything else that I may have missed that you would like to tell me about your experiences with the vaginal tablet?

Thank you for your input and time. We appreciate what we have learnt from you. The end time of interview is_____________ [Turn off recorder]
