# Supplementary material for: Bacterial Vaginosis [BV] knowledge, attitudes and behavioural changes after BV diagnosis among women enrolled in a clinical trial in Boston, USA and Vulindlela, South Africa: a qualitative study
Source: BMC Womens Health. 2025 Dec 12;26:32. doi: 10.1186/s12905-025-04210-3 (PMC12817389; doi:10.1186/s12905-025-04210-3)
Supplement: Supplementary file 5 — Supplementary Material 5. [file 12905_2025_4210_MOESM5_ESM.pdf]

Visit Code [ ] . [ ][ ] [ ]
Phase Visit Interim #

Subject ID 0 6 8 - [ ][ ] - [ ][ ][ ][ ]
Study Number Site Subject Number

Visit Date [ ][ ] [ ][ ][ ] [ ][ ]
dd MMM yy

Baseline behaviour questionnaire

Section A: Obstetrics and gynaecology history

Menstrual history:

1.When did you begin suppression of your periods with hormonal contraception?

- [ ] Just for the study → **Skip to item 2**
- [ ] Before hearing about the study

1a. When did you start suppressive hormonal contraception? [ ][ ][ ] MMM [ ][ ] yy

2. When was the first day of your most recent menses/period?

[ ][ ] dd [ ][ ][ ] MMM [ ][ ] yy ***OR*** [ ] Have not had period in more than 6 months → ***Skip to item 4***

3. When you are menstruating (on your period) what do you use for menstrual protection? (***Please select all that apply***)

- [ ] Sanitary pads (disposable)
- [ ] Sanitary pads (cloth/reusable)
- [ ] Tampons (with and without applicator)
- [ ] Menstrual cups (i.e., Diva cup, flex disc, soft cup, etc.)
- [ ] Period underwear (i.e.thinx)
- [ ] Tissues
- [ ] Cloth
- [ ] Other please specify:________________

Pregnancy history:

4. Have you ever been pregnant? [ ] Yes [ ] No [ ] Don't Know

(No / Don't Know) → **Skip to item 5**

If **yes:**

4a. How many pregnancies have you had? [ ][ ]

4b. How many of these pregnancies were unplanned (by accident)? [ ][ ]

4c. How many deliveries have you had? [ ][ ]

Vaginal Health

5. Have you ever been diagnosed with bacterial vaginosis before today?

- [ ] Yes → **Skip to item 5b**
- [ ] No
- [ ] Don't Know

5a. If no or don't know, how many times in the past year have you been treated for vaginal symptoms? [ ][ ]

5b. If yes, how many times in the past year have you had bacterial vaginosis? [ ][ ]

Subject ID 0 6 8 - ☐☐ - ☐☐☐☐
Study Number Site Subject Number

Baseline behaviour questionnaire

6. What have you used for treatment of vaginal symptoms or BV?

6 a. Antibiotics used:
(Please select all that apply)

- ☐ Metronidazole (*i.e., Flagyl, Metrogel-Vaginal, etc.)*
- ☐ Clindamycin (*Cleocin, Clindesse, etc.*)
- ☐ Tinidazole (*Tindamax*)
- ☐ Secnidazole (*Solosec*)
- ☐ Azithromycin
- ☐ Doxycyline
- ☐ Antibiotic injection
- ☐ Do not know
- ☐ None

6 b. Other treatment used:
(Please select all that apply)

- ☐ Vaginal cream/pessary
- ☐ Home remedies; *please specify:* ______________________________
- ☐ Oral probiotics
- ☐ Vaginal probiotics
- ☐ Boric acid suppositories
- ☐ Herbal medicine; *please specify:* ______________________________
- ☐ Other; *please specify:* ____
- ☐ None

7. Have you ever been diagnosed with one of the following STIs?
(***Please select all that apply***)

- ☐ Gonorrhea
- ☐ Chlamydia
- ☐ Trichomonas
- ☐ Herpes
- ☐ Genital warts
- ☐ HPV
- ☐ Syphilis
- ☐ None of the above
- ☐ I don’t know

Baseline behaviour questionnaire

Sexual/Relationship history

8. What is your current relationship status?
   (***Select all that apply***)
   - ☐ Married or in a stable/committed relationship and living together
   - ☐ Married or in a stable/committed relationship but living separately
   - ☐ Have a casual sexual partner(s)
   - ☐ Not in a sexual relationship
   - ☐ Other, Specify: ____________________
   - ☐ Prefer not to answer

9. How many sexual partners have you had in your lifetime? ☐☐ partners

10. How many sexual partners have you had in the past month? ☐☐ partners

11. Have you ever been in a relationship where you were hit, punched, kicked, assaulted or harmed?

    ☐ Yes ☐ No ☐ Prefer not to answer

12. Are you currently in a relationship where you are being harmed?

    ☐ Yes ☐ No ☐ Prefer not to answer

Baseline behaviour questionnaire

Life Experiences

The questions in this scale ask you about your feelings and thoughts ***during the last month****.*
In each case, you will be asked to indicate by checking an X in the number that corresponds to how often you felt or thought a certain way.

0 = Never	1 = Almost Never	2 = Sometimes	3 = Fairly Often	4 = Very

13a	In the last month, how often have you been upset because of something that happened unexpectedly?	☐ 0	☐ 1	☐ 2	☐ 3	☐ 4
13b	In the last month, how often have you felt that you were unable to control the important things in your life?	☐ 0	☐ 1	☐ 2	☐ 3	☐ 4
13c	In the last month, how often have you felt nervous and "stressed"?	☐ 0	☐ 1	☐ 2	☐ 3	☐ 4
13d	In the last month, how often have you felt confident about your ability to handle your personal problems?	☐ 0	☐ 1	☐ 2	☐ 3	☐ 4
13e	In the last month, how often have you felt that things were going your way?	☐ 0	☐ 1	☐ 2	☐ 3	☐ 4
13f	In the last month, how often have you found that you could not cope with all the things that you had to do?	☐ 0	☐ 1	☐ 2	☐ 3	☐ 4
13g	In the last month, how often have you been able to control irritations in your life?	☐ 0	☐ 1	☐ 2	☐ 3	☐ 4
13h	In the last month, how often have you felt that you were on top of things?	☐ 0	☐ 1	☐ 2	☐ 3	☐ 4
13i	In the last month, how often have you been angered because of things that were outside of your control?	☐ 0	☐ 1	☐ 2	☐ 3	☐ 4
13j	In the last month, how often have you felt difficulties were piling up so high that you could not overcome them?	☐ 0	☐ 1	☐ 2	☐ 3	☐ 4

CAPRISA 068 Plate #012

Visit Code ☐.☐☐ ☐
Phase Visit Interim #

Subject ID 0 6 8 - ☐☐ - ☐☐☐☐
Study Number Site Subject Number

Baseline behaviour questionnaire

Food security

Please indicate whether the statements below were OFTEN, SOMETIMES, or NEVER true for (you/you and the other members of your household) in the last 12 months.

14. The food that (I/we) bought just didn't last, and (I/we) didn't have money to get more.
    - ☐ Often true
    - ☐ Sometimes true
    - ☐ Never true
    - ☐ Prefer not to answer

15. (I/we) couldn't afford to eat balanced meals.
    - ☐ Often true
    - ☐ Sometimes true
    - ☐ Never true
    - ☐ Prefer not to answer

16. In the last 12 months, since (*date 12 months ago*) did (you or other adults in your household) ever cut the size of your meals or skip meals because there wasn't enough money for food?

    ☐ Yes ☐ No ☐ Prefer not to answer

    16a. If Yes, How often did this happen?
    - ☐ Almost every month
    - ☐ Some months but not every month
    - ☐ Only 1 or 2 months
    - ☐ Prefer not to answer

17. In the last 12 months, did you ever eat less than you felt you should because there wasn't enough money to buy food?

    ☐ Yes ☐ No ☐ Prefer not to answer

18. In the last 12 months, were you ever hungry but didn't eat because you couldn't afford enough food?

    ☐ Yes ☐ No ☐ Prefer not to answer

Date: ☐☐ ☐☐☐ ☐☐
dd MMM yy

Completed by: ☐☐☐
